# Distinct effects of PTST2b and MRC on starch granule morphogenesis in potato tubers

**DOI:** 10.1111/pbi.14505

**Published:** 2024-12-10

**Authors:** Anton Hochmuth, Matthew Carswell, Aaron Rowland, Danielle Scarbrough, Lara Esch, Nitin Uttam Kamble, Jeffrey W. Habig, David Seung

**Affiliations:** ^1^ John Innes Centre Norwich Research Park Norwich NR4 7UH UK; ^2^ Simplot Plant Sciences J. R. Simplot Company Boise Idaho 83707 USA

**Keywords:** flow cytometry, granule morphology, potato, starch, starch granule, tubers

## Abstract

The molecular mechanisms underpinning the formation of the large, ellipsoidal starch granules of potato tuber are poorly understood. Here, we demonstrate the distinct effects of PROTEIN TARGETING TO STARCH2b (PTST2b) and MYOSIN RESEMBLING CHLOROPLAST PROTEIN (MRC) on tuber starch granule morphology. A gene duplication event in the *Solanaceae* resulted in two PTST2 paralogs (PTST2a and PTST2b). PTST2b is expressed in potato tubers, and unlike PTST2a, it had no detectable interaction with STARCH SYNTHASE 4. MRC expression was detectable in leaves, but not in tubers. Using transgenic potato lines in the variety Clearwater Russet, we demonstrate that MRC overexpression leads to the formation of granules with aberrant shapes, many of which arise from multiple initiation points. Silencing PTST2b led to the production of striking near‐spherical granules, each arising from a single, central initiation point. Contrary to all reported PTST2 mutants in other species, we observed no change in the number of granules per cell in these lines, suggesting PTST2b is specifically involved in the control of starch granule shape. Starch content and tuber yield per plant were not affected by PTST2b silencing, but MRC overexpression led to strong decreases in both parameters. Notably, the spherical granules in PTST2b silencing lines had a distinctively altered pasting profile, with higher peak and final viscosity than the wild type. Thus, PTST2b and MRC are promising target genes for altering starch granule size and shape in potato tubers, and can be used to create novel starches with altered physicochemical and/or functional properties.

## Introduction

The starch‐rich tubers of potato plants (*Solanum tuberosum* L.) are a staple source of dietary carbohydrates in many parts of the world. Starch is the major storage carbohydrate in plants and is unique amongst carbohydrate polymers in that it forms semi‐crystalline, insoluble granules (Smith and Zeeman, 2020; Zeeman *et al*., 2010). These granules are synthesized in plastids and are composed of two glucose polymers: amylopectin and amylose. Amylopectin is a highly branched polymer that forms the semi‐crystalline granule matrix, whilst amylose is a primarily linear polymer that typically constitutes 17%–21% of potato starch (Seung, 2020). Starch granules of potato tubers are unique amongst major crops in that they are very large (their average diameter is more than double that of maize starch granules) and have a distinct ellipsoid shape (Jane *et al*., 1994).

Granule morphology is thought to be a major factor influencing the physicochemical and nutritional properties of starch, as well as processing quality (Chen *et al*., 2021; Li *et al*., 2021; Lindeboom *et al*., 2004). Large granules have high swelling power during cooking, a characteristic that is associated with the high viscosity of potato starch (Lindeboom *et al*., 2004). However, its high swelling power makes potato starch unsuitable for many extruded products (Liu, 2005). Small granules are also useful in industrial applications like papermaking and biodegradable plastics, as they bind the matrix more tightly due to their increased surface area and they disperse more evenly than larger granules (Lindeboom *et al*., 2004). In vitro work on intact potato starches demonstrated that large granules are digested less efficiently than smaller granules, due to the reduced surface area available for enzymatic digestion (Dhital *et al*., 2010). Therefore, developing methods to specifically manipulate starch granule morphology is important for creating starches with modified properties. However, unlike the good knowledge of the biochemical steps required to synthesize amylopectin and amylose, we are only beginning to understand the molecular factors that determine starch granule morphology, which restricts approaches to engineer granule shape and size.

Recent work, mostly in Arabidopsis leaves, has discovered and characterized proteins involved in granule initiation and morphogenesis, but the relevance of these proteins to the unique morphology of potato tuber granules is not known. Granules of Arabidopsis leaves, like most other leaf starches, have a flattened shape that results from anisotropic granule growth (Burgy *et al*., 2021). The glucosyltransferase, STARCH SYNTHASE 4 (SS4) is required for this anisotropic growth, as mutants deficient in this enzyme produce spherical granules resulting from isotropic growth (Burgy *et al*., 2021; Crumpton‐Taylor *et al*., 2013; Lu *et al*., 2018; Roldán *et al*., 2007). SS4 is also involved in granule initiation, as *ss4* mutants have severely reduced numbers of starch granules in chloroplasts (Mérida and Fettke, 2021; Seung and Smith, 2019).

In addition to SS4, PROTEIN TARGETING TO STARCH2 (PTST2) is a key player that controls starch granule initiation – not only in Arabidopsis but also in cereal endosperm (Chia *et al*., 2020; Matsushima *et al*., 2023; Peng *et al*., 2014; Seung *et al*., 2017). PTST2 interacts with a variety of starch biosynthetic enzymes, including SS4 in Arabidopsis (Seung *et al*., 2017); SS4, ISOAMYLASE 1 (ISA1) and GRANULE‐BOUND STARCH SYNTHASE1 (GBSS1) in rice (Peng *et al*., 2014; Zhang *et al*., 2021); and the ⍺‐GLUCAN PHOSPHORYLASE (PHS1/PHO1) in wheat (Kamble *et al*., 2023). In addition, there are non‐enzymatic proteins that associate with SS4 and PTST2 in Arabidopsis, including MYOSIN RESEMBLING CHLOROPLAST PROTEIN (MRC) (Seung *et al*., 2018; Vandromme *et al*., 2019), MAR1‐BINDING FILAMENT PROTEIN (MFP1) (Seung *et al*., 2018) and a non‐catalytic starch synthase, STARCH SYNTHASE 5 (SS5) (Abt *et al*., 2020). These ‘granule initiation proteins’ (PTST2, MRC, MFP1 and SS5) are proposed to act in granule initiation in Arabidopsis leaves by modulating SS4 activity, location and substrate availability, but they are unlikely to have a direct role in granule morphogenesis. Arabidopsis mutants deficient in any of these five proteins have reduced numbers of starch granules within each chloroplast, resulting in larger granules, but the granules retain their flattened morphology and irregular shape like the wild type (Abt *et al*., 2020; Seung *et al*., 2017, 2018).

Early studies using transmission electron microscopy suggest that potato amyloplasts typically contain one large granule (Kram *et al*., 1993; Ohad *et al*., 1971). It is possible that the ellipsoid granule morphology results from the physical constraints imposed by the amyloplast, and/or from controlled deposition of polymers mediated by specific proteins during granule growth. Indeed, a recent study reported that potato mutants lacking PHO1a (the dominant PHO1 isoform in tubers) have smaller, rounder starch granules than the wild type – suggesting the requirement of specific proteins for correct granule morphology (Sharma *et al*., 2023). Here, we discovered that PTST2b, the dominant PTST2 isoform in potato tubers, does not influence granule size or the number of granules initiated, but plays an essential role in controlling granule shape. Potato tubers also have no detectable MRC expression, but when ectopically overexpressed in the tuber, it leads to the formation of irregularly shaped starch granules – many of which originate from multiple initiation points. We therefore provide new insights into the diverse roles of initiation proteins, the control of granule shape in potato, and their implications on starch physicochemical properties.

## Results

### Potato tubers express a unique complement of granule initiation proteins

**Figure 1:**
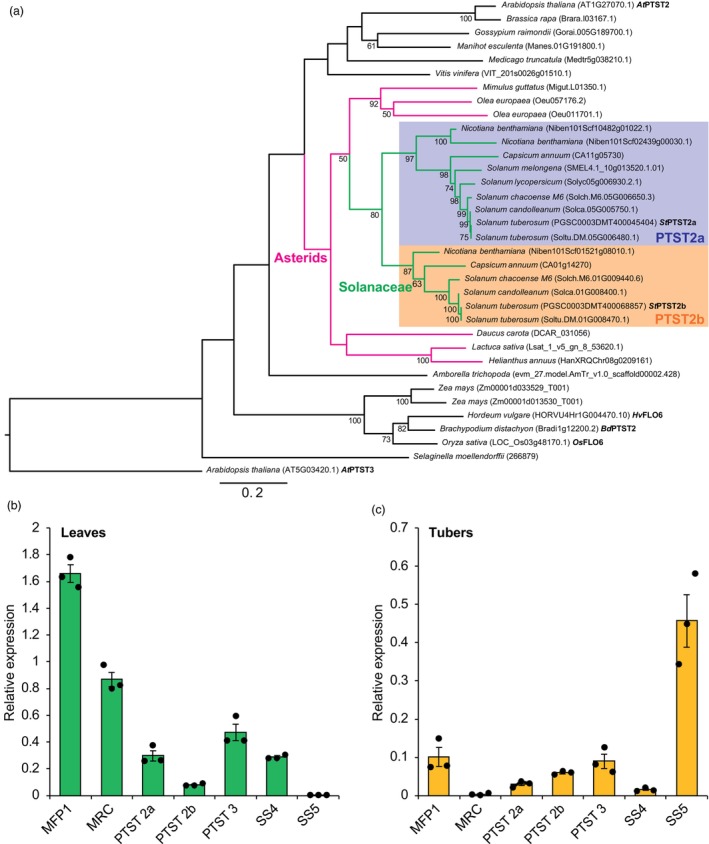
PTST2a and PTST2b result from gene duplication in the Solanaceae and are expressed in potato tubers. (a) A maximum likelihood phylogenetic tree was produced using RaxML from PTST2 sequences with 1000 bootstrap replicates. Bootstrap values >50 are shown next to the nodes. Branches corresponding to Solanaceae sequences are shown with green branches, and those corresponding to other Asterids are shown in magenta. The PTST2a clade is shaded in blue, whilst the PTST2b clade is shaded in orange. Branch lengths represent the number of substitutions per site, indicated by the scale bar. (b and c) Expression levels of granule initiation protein orthologs in potato leaves (b) and tubers (c). Expression values were quantified by RT‐qPCR and are expressed relative to the reference gene, Sec3. Values represent means ± SEM from *n* = 3 biological replicates (each replicate represents a separate plant).

We used phylogenetic tree analyses to identify the potato orthologs of Arabidopsis granule initiation proteins (Table S1; Figure S1). We used sequences that were homologous to Arabidopsis granule initiation proteins (SS4, PTST2, PTST3, MRC, MFP1 and SS5) identified using BLASTp in both versions of the potato genome (v4.03 and v6 from the doubled monoploid Phureja DM1‐3 genome) (Pham *et al*., 2020; Potato Genome Sequencing *et al*., 2011). We also searched the Phytozome, SolGenomics and SpudDB databases for homologues from other Solanaceae species, as well as diverse species of the Viridiplantae. Potato had orthologs of all six granule initiation proteins. Interestingly, potato and some other Solanaceae species (*Nicotiana benthamiana* and *Capsicum annum*) had two paralogs of PTST2, which we named PTST2a and PTST2b (Figure 1a). Potato PTST2a and PTST2b are located on Chromosomes 5 and 1, respectively, and were also present at these locations in the wild tuber‐bearing potato relatives *Solanum candolleanum* and *Solanum chacoense*. PTST2a and PTST2b sequences formed two distinct clades on the tree that contained only Solanaceae sequences. The fact that PTST2a and PTST2b are found in other Solanaceae species, and reside on different chromosomes in potato, strongly suggests that they are paralogs arising from an early duplication during the divergence of the Solanaceae, rather than being allelic variation at a single PTST2 locus in tetraploid potato. However, the *Solanum melongena* and *Solanum lycopersicum* genomes contained only PTST2a, suggesting that they lost PTST2b. We did not identify any species that solely had PTST2b.

We then used RT‐qPCR to assess transcript levels of the initiation proteins in both leaves and tubers of a commercial variety, Clearwater Russet (Figure 1b,c). Transcripts of most genes were detected in both leaves and tubers. The two exceptions were MRC, with high transcript levels in leaves and undetectable transcripts in tubers, and SS5, which by contrast had high transcript levels in tubers but undetectable transcripts in leaves. Since MRC promotes starch granule initiation in Arabidopsis leaf chloroplasts (Seung *et al*., 2018; Vandromme *et al*., 2019), we hypothesized that the low expression of MRC could underpin the low numbers of starch granules per amyloplast in tubers (Ohad *et al*., 1971). Transcripts for both PTST2 paralogs were detected in leaves and tubers (Figure 1b,c). However, leaves had almost four times more PTST2a transcripts than PTST2b. These findings were consistent with data from a public gene expression database for potato leaves and three stages of tuber development (stolon, young and mature stage) (Massa *et al*., 2011) (Table S1). Overall, we demonstrate that a unique complement of granule initiation proteins is expressed in potato tubers, which includes PTST2b and lacks MRC.

### PTST2b is an atypical PTST2 isoform that does not interact with SS4

**Figure 2:**
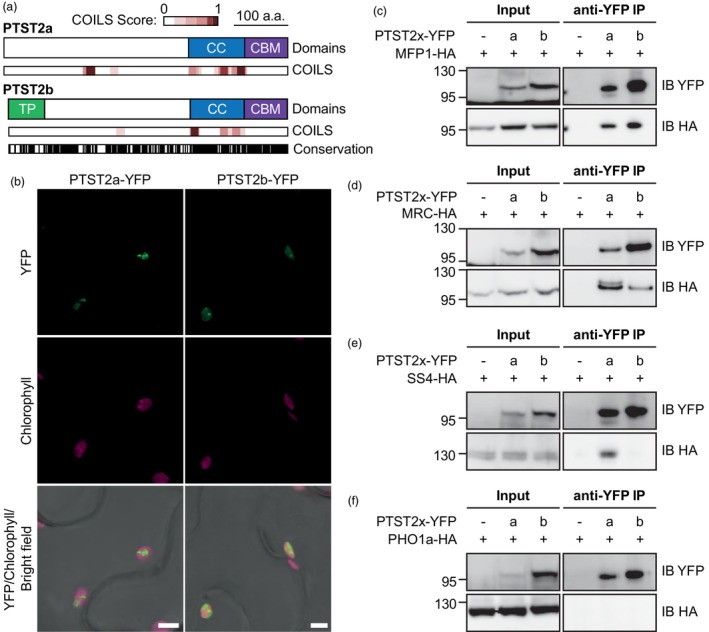
PTST2a and PTST2b differ in protein–protein interaction partners. (a) Schematic illustration of domains in PTST2a and PTST2b. The plastid transit peptide (TP), coiled coil domains (CC) and Carbohydrate Binding Module 48 (CBM) domains are shown. The locations of coiled coils predicted by the COILS program with a 14‐amino acid window are shown with the probability score (from 0 to 1, where 1 is the highest probability). The degree of sequence conservation from a pairwise alignment of PTST2a and PTST2b is illustrated underneath, where the location of identical amino acids are marked in black. (b) Plastidial localization of PTST2a‐YFP and PTST2b‐YFP when transiently expressed in *Nicotiana benthamiana* leaves, observed with confocal laser‐scanning microscopy. Bars = 5 μm. (c–f) Pairwise immunoprecipitations using PTST2a‐YFP and PTST2b‐YFP as bait proteins, against MFP1‐HA (c), MRC‐HA (d), SS4‐HA (e) and PHO1a‐HA (f) prey proteins. Immunoprecipitations were conducted with anti‐YFP beads. The presence of the bait and prey proteins in the Input and immunoprecipitates (anti‐YFP IP) were detected using immunoblots (IB) with YFP and HA antibodies.

To further investigate how PTST2a and PTST2b differ from each other, we first compared their amino acid sequences. The full amino acid sequences of potato PTST2a and PTST2b shared 58% identity and 70% similarity under BLASTp alignment, and their coding sequences at the DNA level showed 74% identity after a ClustalW alignment (Figure S2). Both paralogs had the typical CBM48 domain at the C‐terminal end, which shared 80% identity and 90% similarity (Figure 2a). Directly preceding the CBM48 domain of both paralogs was a predicted coiled‐coil domain, which was more variable in sequence between the paralogs (63% identity and 76% similarity) compared to the CBM48. PTST2a also had strong coiled coil predictions towards the N‐terminal end of the protein. Interestingly, the TargetP program for predicting subcellular localization (Almagro Armenteros *et al*., 2019) could only predict a plastid transit peptide for PTST2b. Despite this, we experimentally confirmed that both PTST2 paralogs were plastidial by transiently expressing fused versions to a C‐terminal YFP tag in *N. benthamiana* (Figure 2b).

Coiled coils are generally implicated in protein–protein interactions (Mason and Arndt, 2004). Given the variability in the coiled coil regions between PTST2a and PTST2b, we compared the ability of these isoforms to interact with its partner proteins using pairwise immunoprecipitations with proteins heterologously expressed in *Nicotiana* leaves (Figure 2c–f). Like Arabidopsis PTST2, both PTST2 paralogs could interact with MFP1 and MRC; and unlike the PTST2 ortholog of wheat, neither PTST2a nor PTST2b could interact with PHO1a in this assay. Most strikingly, under these assay conditions, we could not detect an interaction between PTST2b and SS4, whereas robust interaction was detected for PTST2a and SS4. This difference in binding partner specificity and restricted phylogenetic occurrence suggests PTST2b is unique amongst characterized PTST2 proteins and deserves further investigation.

### Size distribution of tuber starch granules is altered in MRC overexpression lines but not in PTST2b silencing lines

Our analyses above revealed two unique features of the complement of granule initiation proteins in potato tubers: 1) the presence of the unique PTST2b paralog and 2) the absence or very low expression of MRC. To investigate the significance of these features on starch granule number and morphology, we created constitutive silencing lines of PTST2b using RNA interference (siPTST2b lines) by expressing an inverted repeat of the PTST2b sequence under a 35S promoter; and constitutive MRC overexpression lines (MRC‐OE lines) to induce MRC expression during tuber development using the 35S promoter. All lines were generated in Clearwater Russet. Lines transformed with an empty vector (CW Empty) were isolated as controls.

**Figure 3:**
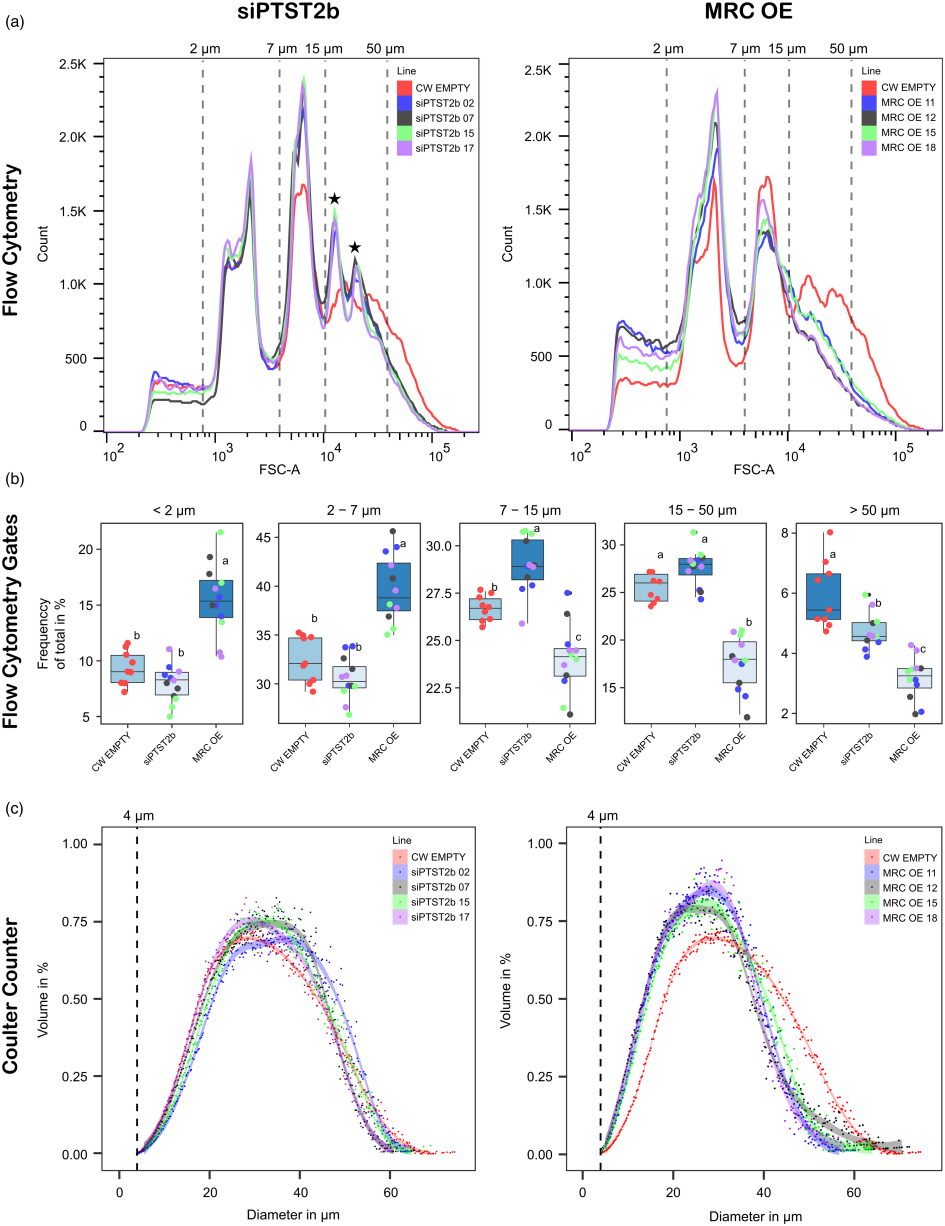
Starch granule size distributions in PTST2‐RNAi and MRC‐OE transgenic lines. (a) Purified starch granules were analysed on the flow cytometer. Representative forward scatter (FSC) traces from three independent biological replicates (using tubers harvested from separate plants) are shown. (b) Quantification of distributions using flow cytometry gates on the FSC data from a. Data are plotted by construct, incorporating data from all lines (lines are distinguished by the same colour key as a). The bottom and top of the box represent the lower and upper quartiles, respectively, and the band inside the box represents the median. The whiskers represent the minimum and maximum values that are within 1.5× of the interquartile range. Values marked with different letters are significantly different under a one‐way ANOVA (*P* < 0.05). The data from individual lines can be found in Figure S5. (c) Coulter counter analysis of granule size distribution (by volume). Jitter points represent the mean from three biological replicates. The smoothed trendline was created using a general additive model (GAM) using the mean data points, where the width of the ribbon represents the 95% confidence interval of the model.

Tubers from initial transformants for siPTST2b and MRC‐OE were cultivated in the glasshouse (12 independent lines for each), and were screened for alterations in granule size and/or shape simultaneously using a flow cytometer (Thieme *et al*., 2023). The distribution of starch granules (by number) was plotted against the forward scatter (FSC), which primarily correlates with granule size but is also affected by major changes in particle shape (Mage *et al*., 2019; Thieme *et al*., 2023). Some of the siPTST2b lines had strongly altered FSC traces compared to the wild type, whilst others did not (Figure S3). Four selected lines with strong alterations in FSC trace within this first generation (siPTST2b #2, #7, #15 and #17) were propagated for the second generation in the glasshouse, on which we repeated the flow cytometer analysis. The empty vector control had a broad FSC trace, with most granules falling within a range corresponding to 2–50 μm according to size standards (Figure 3a). There were several distinct peaks within the trace corresponding to 4, 11, 23 and 40 μm; suggesting that potato starch could be composed of subpopulations of starch granules with distinct sizes and/or shapes. The trace for the empty vector control (analysed in the second generation) was similar to that of the untransformed Clearwater Russet (analysed in the first generation) (Figure S3). The siPTST2b lines had a distinctive FSC trace, where the peaks at 23 and 40 μm were sharper than in the control, and slightly shifted to the smaller sizes (indicated with stars) (Figure 3a,b). This trace was identical in all four selected siPTST2b lines and highly reproducible between the two glasshouse‐grown generations (Figure 3A; Figure S3). Interestingly, Coulter counter analyses of the same samples suggested that the observed alterations in the FSC for siPTST2b lines were mainly due to changes in granule shape, rather than changes in granule size. By contrast to flow cytometry, the Coulter counter quantifies granule size by volume and is less sensitive to changes in granule shape, and the volumetric size distributions of the four selected siPTST2b lines were largely identical to that of the control (Figure 3c). It is therefore unlikely that silencing PTST2b affects starch granule size, but likely that it affects granule shape.

Unlike siPTST2b lines, we observed a true decrease in granule size in the selected MRC‐OE lines. Screening all lines in the first glasshouse generation using the flow cytometer revealed lines with strong shifts towards the smaller size range in the forward scatter (Figure S4). We selected four lines with the strongest alterations (MRC OE #11, #12, #15 and #18) for re‐analysis in a second generation. The FSC trace was highly reproducible in this second generation: with no clear peaks corresponding to 15–50 μm and significantly smaller granules (Figure 3a). MRC OE lines had 20%–50% more granules in the smaller size categories (<2 μm and 2–7 μm) compared with the control, and about 12%–45% significantly fewer granules in the mid‐ and large‐size categories (7–15, 15–50 and > 50 μm) (Figure 3B; Figure S5). This strong shift towards smaller granules was confirmed using the Coulter counter in all selected MRC‐OE lines. This indicates that MRC‐OE lines have genuinely smaller granules (Figure 3c).

### Granule morphology is drastically altered in PTST2b silencing and MRC overexpression lines

**Figure 4:**
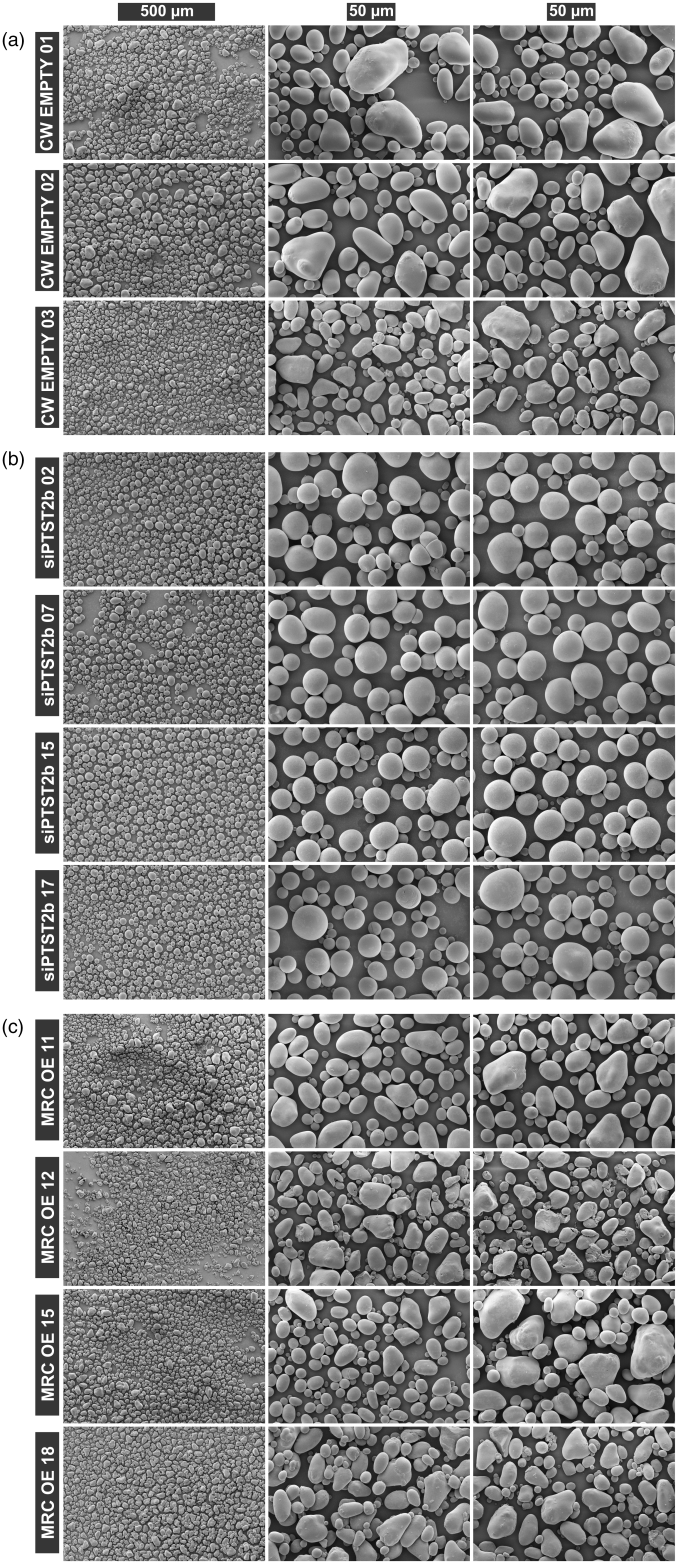
Starch granule morphology of siPTST2b and MRC‐OE transgenic lines. Purified starch granules were imaged using scanning electron microscopy, for (a) CW Empty vector controls, (b) siPTST2b lines and (c) MRC‐OE lines. For each line, one low‐magnification image and two high‐magnification images (from different regions of the same sample) are shown.

Our combined flow cytometry and Coulter counter analyses suggested that PTST2b silencing likely affects granule shape, and whilst MRC overexpression affects granule size, we could not draw conclusions on granule shape. Therefore, to explore starch granule morphology in our transgenic lines in detail, we analysed purified starch granules by scanning electron microscopy (SEM). Strikingly, granules in the four selected siPTST2b lines appeared almost perfectly spherical, rather than having the typical ovoid morphology observed in the empty vector control (Figure 4). Granules in the MRC‐OE lines were not only smaller but also seemed more irregular in shape.

To quantify these alterations in granule morphology in our transgenic lines, we developed a novel imaging flow cytometer‐based method for analysing the diversity of starch granule shapes within our samples. We acquired 20 000 images of granules per sample, and after filtering to remove images containing multiple granules and non‐starch particles, 75%–82% of images of individual granules were of sufficient quality to quantify shape parameters (Figure 4; Figure S6). We calculated two shape parameters – circularity and aspect ratio. Circularity is defined as the average length between the centre to the edge of the granule, divided by the variance in this length. More circular and rounded objects have higher circularity values, since there is low radial variance, whereas irregular objects would have lower values. The aspect ratio is defined as the minor axis of the granule divided by the major axis, such that a perfectly round granule would have the maximum aspect ratio of one.

**Figure 5:**
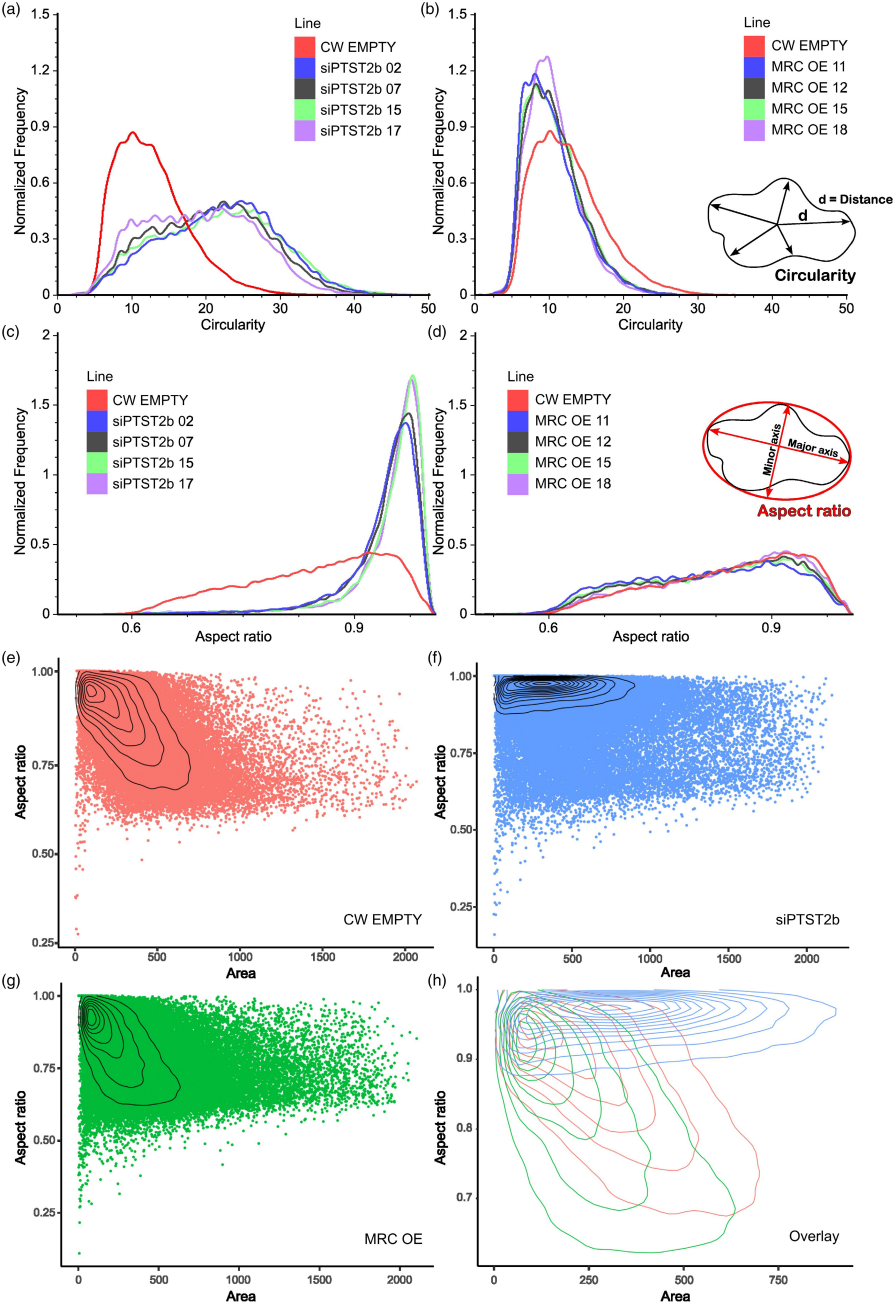
Granule shape parameters from imaging flow cytometry. (a, b) The circularity of starch granules is based on the average distance of the object boundary from its centre divided by the variation of this distance as shown in panel (b). The frequency histogram of granules at each circularity value was plotted for siPTST2b lines (a) and MRC‐OE lines (b). 15 000–17 000 granules were analysed per sample. (c, d) Same as (a)‐(b), except plotting the frequency of granules over the aspect ratio that is calculated by dividing the minor axis by the major axis as shown in panel (d). (e–g) Scatter plots of granule aspect ratio plotted against granule size (area). Granules from all lines analysed in (c) and (d) were pooled to generate these plots. Each point represents a single granule, and the contour lines in the radar plot represent data distribution by indicating regions of equal point density. (h) Overlay of radar plots from e–g to facilitate comparison between genotypes.

In line with our microscopy observations, granules in siPTST2b lines had a very strong shift towards higher circularity values and their aspect ratios were much closer to 1 than granules of the control (Figure 5a,c). By contrast, no changes were seen in the aspect ratio for granules from the MRC‐OE lines, whereas the circularity of these granules had a significant decrease in circularity values (*P*‐value <0.05, see Table S3) – reflecting their increased irregularity in shape (Figure 5b,d). We then assessed the relationship between granule size and shape in our samples. When we plotted the aspect ratio against granule size (the 2‐D area occupied by the granule in the image) for all granules imaged per line, we saw a strong trend that granules became less circular (i.e., had decreased aspect ratios) as their size increased in the control and MRC‐OE lines (Figure 5e,g). By contrast, in the siPTST2b lines, the aspect ratio did not decrease as granule size increased, with most granules maintaining aspect ratios >0.9 (Figure 5f). siPTST2b lines therefore produce spherical granules regardless of granule size, whereas in the control and MRC‐OE lines, only the smaller granules are spherical (Figure 5h).

**Figure 6:**
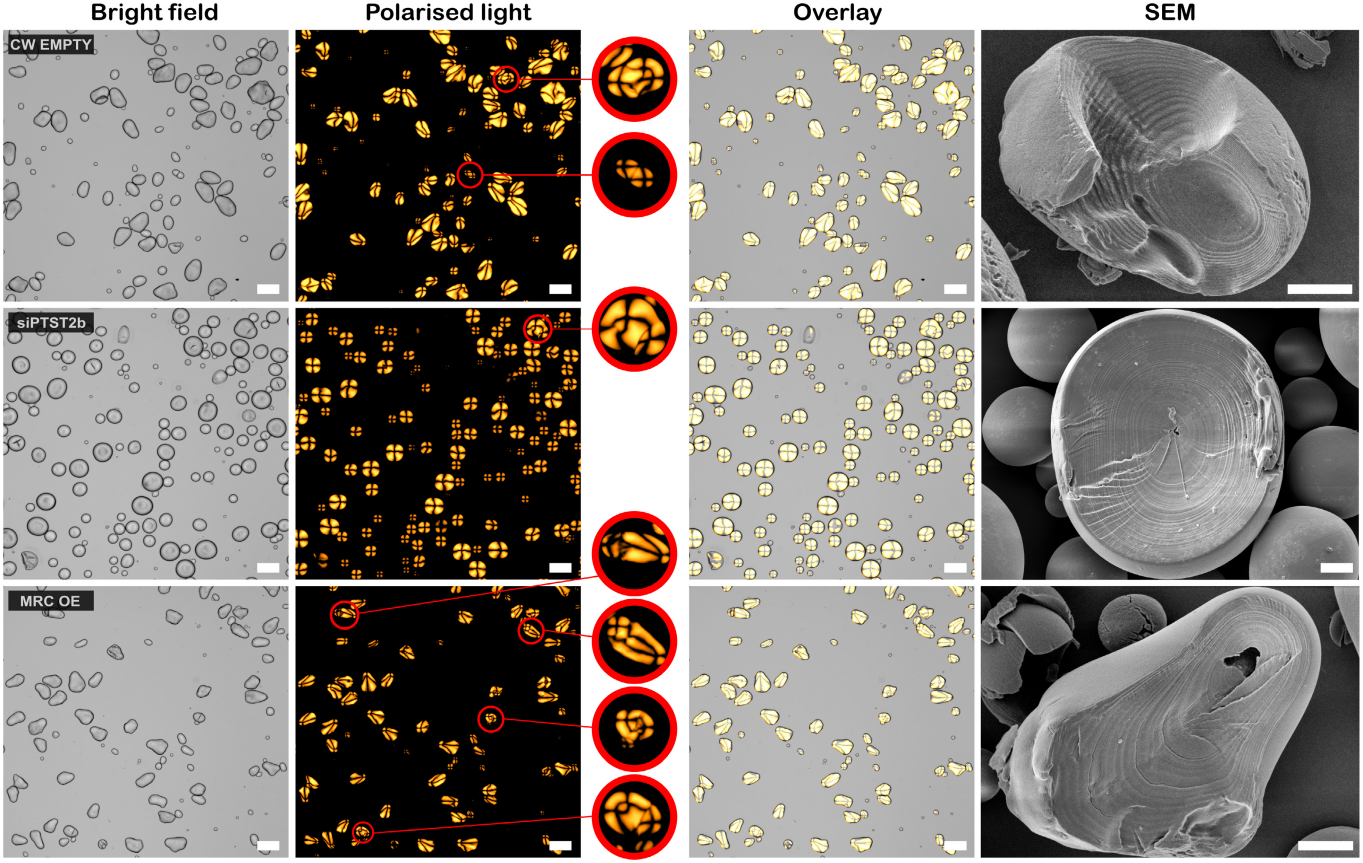
Observation of internal granule organization using polarized light and scanning electron microscopy. Purified starch granules were imaged using standard bright field and cross‐polarized light microscopy. Granules containing multiple initiation points are marked with a red circle, and insets showing a closeup image (3× magnification) are provided. Bars = 50 μm. Scanning electron micrographs (SEM) of fractured starch granules reveal the hilum and growth rings. Bars = 5 μm.

Such effects of PTST2 and MRC on granule shape were not expected since in Arabidopsis, these proteins affect the number of starch granule initiations and granule size, but not granule shape (Seung *et al*., 2018). We therefore examined if the aberrant granule morphology could be attributed to altered granule initiation. We first examined starch granules under polarized light, where the number of initiation points within granules can be visualized as a maltese cross over centres of the crystalline organization (the centre of the cross typically coincides with the granule hilum) (Figure 6). In the control, most starch granules had a single cross that deviated from the centre of the ovoid granule, consistent with anisotropic granule growth from a single hilum. By contrast, in siPTST2b granules, a single cross formed over the centre of the spherical starch granule, indicating a central hilum. Occasionally, granules that had multiple crosses were observed, and their frequency was comparable between PTST2b silencing lines and the controls. In the MRC‐OE line, most granules had single deviated centrepoint (like those of the control), but there was a large proportion of irregularly shaped granules containing multiple crosses. These findings were confirmed by SEM on fractured granules. siPTST2b granules had a central hilum and perfectly concentric growth rings, indicating that they grew isotropically from a single initiation. In the control and MRC‐OE lines, the hilum was displaced from the centre of the granule and surrounded by eccentric growth rings.

**Figure 7:**
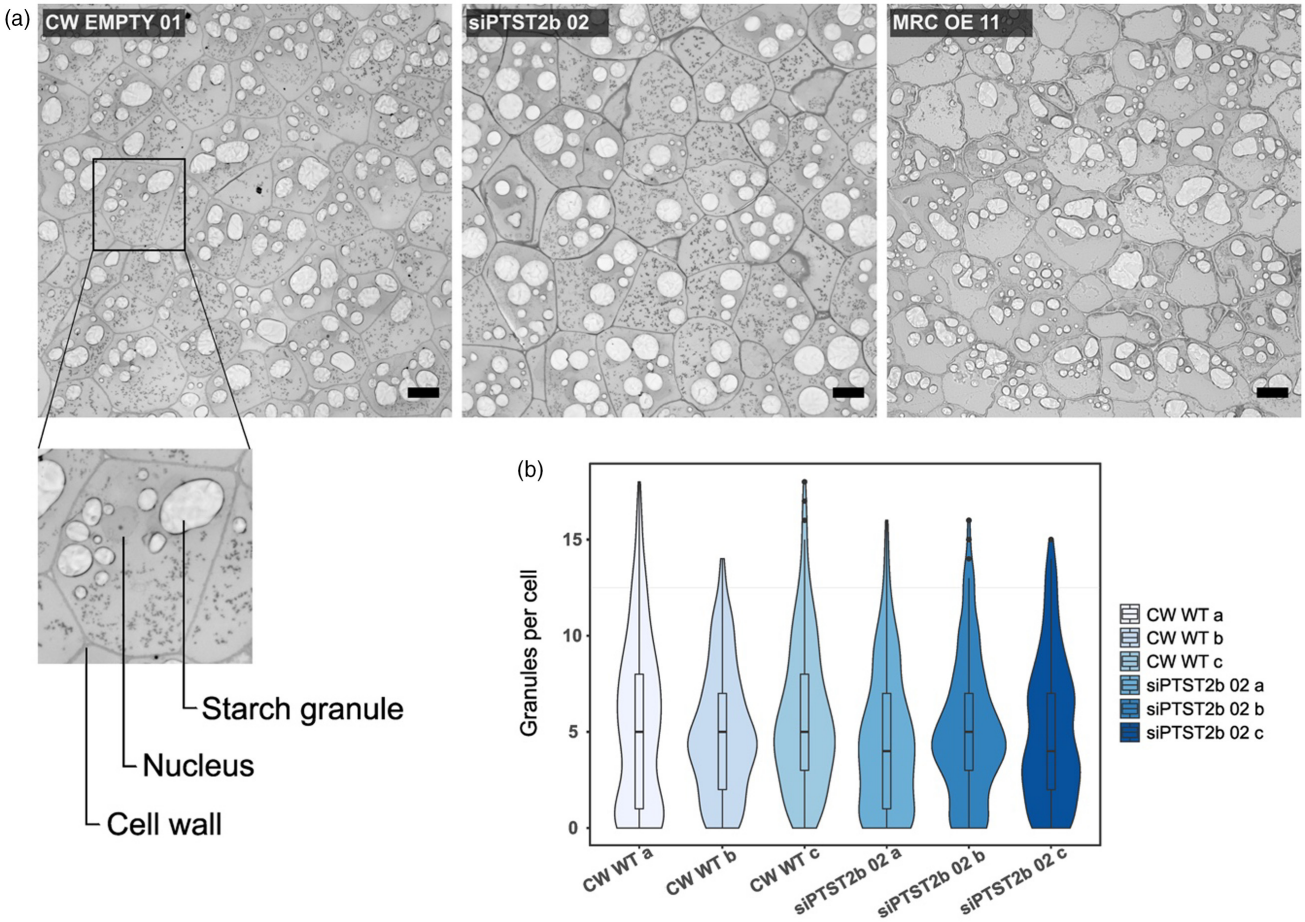
Silencing PTST2b does not alter the number of granules per cell. (a) Starch granules in tuber parenchyma cells visualized using light microscopy. Semi‐thin sections were prepared from fixed, resin‐embedded tuber segments and stained with Toluidine blue before imaging. Bars = 50 μm. (b) Quantification of the number of starch granules per cell. Three replicates (prepared from three individual plants) were analysed, counting 200 cells per replicate. There were no significant differences observed between samples under a one‐way ANOVA at *P* < 0.05.

Finally, to assess starch granule number, we examined the granules in thin sections of the tuber parenchyma tissue using light microscopy. The distinct changes in starch granule shapes in siPTST2b and MRC‐OE lines were visible in these sections (Figure 7a). However, the number of granules per cell was not significantly altered in the siPTST2b lines (Figure 7b).

Overall, our data suggest that PTST2b is not required for correct starch granule number, but is rather required for correct granule morphology in potato tubers. siPTST2b lines produce spherical granules due to the loss of anisotropic granule growth. In contrast, overexpression of MRC appears to increase the frequency of irregular granules, many of which arise from multiple initiations.

### The alteration in starch granule morphology is correlated with the degree of PTST2b silencing or MRC overexpression

**Figure 8:**
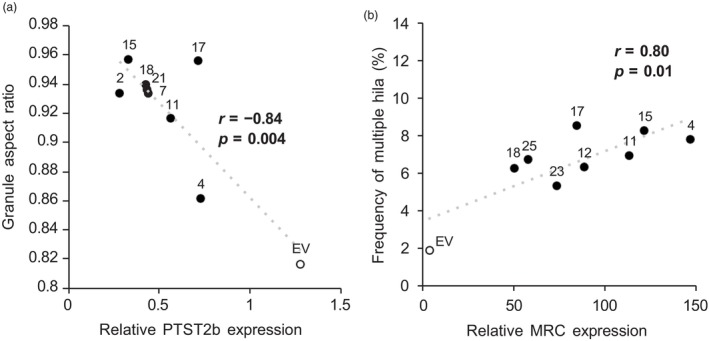
Granule morphology phenotypes correlate to the degree of PTST2b silencing or MRC overexpression. PTST2b and MRC expression in developing tubers were quantified using RT‐qPCR (Figure S7). (a) Granule morphology was quantified for each PTST2b silencing line using the imaging flow cytometer as the average aspect ratio of 18 000 individual granules. (b) The frequency of starch granules with multiple hila was calculated by observing on average 800 individual granules under polarized light microscopy. Values for transgenic lines are shown in filled circles, whilst those for the empty vector control are shown as unfilled circles. The Pearson's correlation coefficient (*r*) and its associated *P*‐value are shown.

To ensure that the changes in granule morphology in our transgenic lines correlated to the degree of silencing or overexpression, we assessed the amount of PTST2b and MRC transcript accumulation using RT‐qPCR in a broader set of transgenic lines (Figure S7), and correlated these values with a quantitative measure of the main phenotypes we observed so far. In PTST2b lines, we associated gene expression levels with the average aspect ratio of granules measured by imaging FC, which demonstrated that the spherical granule phenotype was highly correlated to the degree of PTST2b silencing (Figure 8a). In the MRC‐OE lines, we scored the frequency of granules that were observed to have multiple hila using polarized light microscopy, and we found that the frequency of these granules was correlated with the degree of overexpression (Figure 8b). To ensure our silencing was specific to PTST2b and not PTST2a, we also quantified PTST2a transcripts in our PTST2b lines. Even in lines where PTST2b was strongly silenced, there was no significant reduction in PTST2a transcripts (Figure S8).

### Tuber starch content is reduced in MRC‐OE but not in siPTST2b

**Figure 9:**
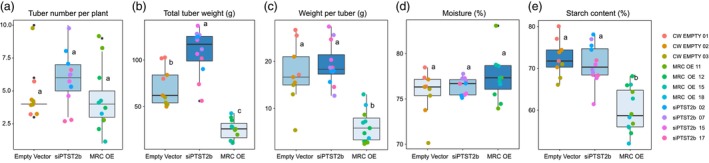
Tuber yield and composition in siPTST2b and MRC‐OE transgenic lines. Three biological replicates (individual plants) per line were grown in the glasshouse. To gain an overview of changes in tuber yield and composition, data are plotted by construct, incorporating data from all lines (lines are distinguished by colour). The bottom and top of the box represent the lower and upper quartiles, respectively, and the band inside the box represents the median. The whiskers represent the minimum and maximum values that are within 1.5× of the interquartile range. Values marked with different letters are significantly different under a one‐way ANOVA (*P* < 0.05). The data from individual lines can be found in Table 1. (a) Tuber yield per plant (by number of tubers). (b) Tuber yield per plant (by total tuber weight). (c) Average weight of single tubers. (d) Moisture content of tubers. (e) Starch content of tubers.

Given these alterations in starch granule shape and size distributions in the transgenic lines, we investigated whether they were accompanied by differences in tuber size or tuber composition. For siPTST2b lines, the total number of tubers produced in the glasshouse was identical to the control lines (Figure 9; Table 1). The total tuber weight per plant for siPTST2b lines was significantly higher than the wild type (Figure 9), but this is unlikely to be a direct consequence of silencing PTST2b since it was not seen in all of the individual lines (Table 1). Further, the average weight of individual tubers was not different between the wild type and siPTST2b (Figure 9). The moisture content and starch content were also not altered in siPTST2b lines. MRC‐OE plants produced similar numbers of tubers per plant, but by contrast to siPTST2b lines, the tubers were approximately half the weight of the controls, resulting in a significant reduction in tuber yield per plant (by weight). Whilst the MRC‐OE tubers had identical moisture content, the starch content was significantly less than the control. Thus, it is likely that the decreased starch content contributes to the smaller granules observed in this line.

**Table 1:** Tuber yield and composition in siPTST2b and MRC‐OE transgenic lines

Line	Tuber number per plant	Total tuber weight (g)	Weight per tuber (g)	Moisture (%)	Starch content (%)
Empty vector 1	4.3 ± 0.9^a^	95.0 ± 7.5^abc^	23.1 ± 3.0^a^	77.3 ± 0.6^a^	76.3 ± 2.4^a^
Empty vector 2	3.7 ± 0.3^a^	65.3 ± 10^cd^	17.7 ± 1.7^ab^	75.7 ± 1.0^a^	72.8 ± 1.3^ab^
Empty vector 3	6.0 ± 2.0^a^	55.3 ± 2.4^cd^	11.1 ± 2.9^abcd^	73.8 ± 1.9^a^	68.3 ± 1.4^abc^
siPTST2b 02	6.7 ± 0.9^a^	113.3 ± 11.7^ab^	17.2 ± 0.7^abc^	75.9 ± 0.6^a^	76.0 ± 1.1^a^
siPTST2b 07	7.0 ± 1.5^a^	122.3 ± 5.2^a^	19.0 ± 3.7^ab^	77.3 ± 0.2^a^	71.9 ± 2.5^abc^
siPTST2b 15	5.7 ± 0.3^a^	122.0 ± 8.7^a^	21.9 ± 2.9^a^	76.1 ± 0.6^a^	66.7 ± 2.8^abcd^
siPTST2b 17	4.3 ± 1.3^a^	77.3 ± 13.4^bc^	19.3 ± 2.9^ab^	76.3 ± 0.4^a^	69.6 ± 1.2^abc^
MRC‐OE 11	6.7 ± 1.9^a^	23.6 ± 1.9^d^	4.6 ± 2.0^cd^	78.1 ± 2.7^a^	60.1 ± 2.2^cd^
MRC‐OE 12	6.0 ± 1.2^a^	28.3 ± 8.2^d^	4.5 ± 0.8^d^	76.2 ± 1.3^a^	62.9 ± 4.2^bcd^
MRC‐OE 15	3.0 ± 1.0^a^	23.3 ± 5.2^d^	9.1 ± 2.0^bcd^	77.6 ± 0.6^a^	61.6 ± 3.0^bcd^
MRC‐OE 18	3.3 ± 0.3^a^	21.7 ± 10.7^d^	6.0 ± 2.4^bcd^	77.6 ± 1.0^a^	55.5 ± 1.8^d^

*Note*: Three biological replicates (individual plants) per line were grown in the glasshouse. Values marked with different letters are significantly different under a one‐way ANOVA (*P* < 0.05).

We also assessed whether starch polymer composition or structure was altered in our transgenic lines. Overall, siPTST2b lines and MRC‐OE lines had a small but significant decrease in amylose content compared to the control (Figure S9a). The amylopectin chain length structure was also slightly altered in both lines compared to the control, with siPTST2b lines having a higher proportion of chains at DP 9–12, and MRC‐OE lines having a higher proportion of longer chains (DP > 15) (Figure S9b,c). Given that these changes in amylose content and amylopectin structure are relatively small, it is unlikely that they cause the altered granule morphology.

### The spherical starch granules in siPTST2b lines have novel physicochemical properties

**Figure 10:**
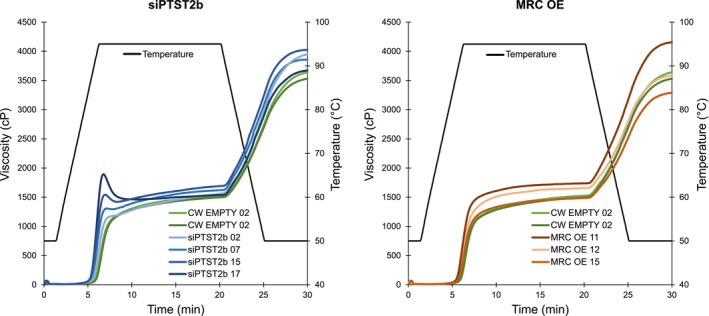
Rapid visco analysis (RVA) of purified siPTST2b and MRC OE starch. Purified starch (1.5 g) was gelatinized in 25 mL of water.

Given the novel morphology of the starches in our transgenic lines, we tested whether they had altered starch physicochemical properties. We explored their gelatinization behaviour using the Rapid Visco Analyser (RVA). The starch from the control and MRC‐OE lines had similar pasting profiles (Figure 10). However, the siPTST2b lines had more prominent peak viscosity and breakdown than the control lines. The peak viscosity correlated to the degree of roundness observed in the granules, whereby the two lines with the most prominent peak viscosities (siPTST2b #15 and #17) were also the lines with the granules had the largest aspect ratio (Figures 5C and 10). The final viscosity after cooling also tended to be higher in the siPTST2b lines compared to the control lines (Figure 10). Thus, altering granule shape by silencing PTST2b influences the pasting profile of starch.

## Discussion

### The novel PTST2b isoform is involved in granule morphogenesis in potato tubers

Potato starch granules have been extensively described as being ellipsoid/ovoid in shape, but the origins of this trait were not understood (Hall and Sayre, 1969; Jane *et al*., 1994). The control of granule shape is poorly understood in any species, but since the shape of potato starch granules appears relatively simple in comparison to the flattened or polygonal granules found in other species (e.g., wheat and rice), granule growth mechanisms in potato tubers have received very little attention. Our results demonstrate that the ovoid shape of these granules results from active morphogenesis mediated by PTST2b. If PTST2b is deficient, the tuber starch granules grow isotropically into spheres (Figures 4 and 5).

We present several lines of evidence that PTST2b is an atypical PTST2 isoform in terms of its occurrence, properties and function. Firstly, PTST2b is phylogenetically restricted to Solanaceae because the isoform arose from a duplication within that family, and some species have subsequently lost it (Figure 1). In contrast, all the Solanaceae species we examined had retained PTST2a. Secondly, PTST2a and PTST2b have distinct specificities for protein‐binding partners. PTST2a can interact with SS4, like the PTST2 isoforms described in Arabidopsis and rice (Seung *et al*., 2017; Zhang *et al*., 2021) (Figure 2). In contrast, we could not detect an interaction between PTST2b and SS4. Finally, unlike all other PTST2 orthologs examined so far, PTST2b affects the shape of granules but not the number of granules initiated or granule size (Figures 4, 5, 6, 7). The spherical granules in the siPTST2b lines had a similar volume to wild‐type granules (Figure 3) and had a single initiation point (Figure 6). Also, the number of granules per cell was the same between the wild‐type and the silencing line (Figure 7). This is opposite to Arabidopsis *ptst2* mutants, which had fewer but larger granules compared to the wild type, but still retained the flattened shape of typical wild‐type starch granules (Seung *et al*., 2017, 2018; Thieme *et al*., 2023). In the endosperm of barley, wheat and *Brachypodium*, deficiency in PTST2 (FLO6/BGC1) results in a range of phenotypes associated with granule number, such as the lack of small B‐type granules or the initiation of multiple granules per amyloplast that fuse into compound granules (Chia *et al*., 2020; Saito *et al*., 2017; Suh *et al*., 2004; Watson‐Lazowski *et al*., 2022). In rice, *flo6* mutants have general defects in compound granule formation, including some amyloplasts that contain a single granule (Peng *et al*., 2014; Zhang *et al*., 2021). An exclusive role for PTST2b in granule morphogenesis is so far a unique observation in potato tuber, and demonstrates that PTST2 orthologs can have roles beyond granule initiation.

It is important to note that both PTST2a and PTST2b are expressed in tubers. Transcript levels of PTST2a did not change in the siPTST2b lines (Figure S8), so it is unlikely that there is unspecific silencing of PTST2a or any compensatory mechanism that increases PTST2a expression after PTST2b is silenced. The role of PTST2a in potato tubers remains to be determined, but it is unlikely to play a redundant role in granule morphogenesis with PTST2b. The granules in siPTST2b lines already had near maximum roundness (aspect ratio close to 1 – Figure 5), so the presence of PTST2a is clearly insufficient to mediate normal granule morphogenesis. It is possible that PTST2a may have a more canonical PTST2 role in controlling the number of granule initiations. This could be tested by creating and analysing PTST2a silencing or knockout lines.

It is tempting to speculate that differences in PTST2a and PTST2b function could have been driven by the different structural and biochemical environments in which granule formation occurs between tubers and leaves. Amyloplasts in the tuber are structurally very different from chloroplasts in leaves, which could lead to inherent differences in the mechanisms of granule initiation and morphogenesis. They also potentially differ in their biochemistry as our gene expression data shows differential expression of the other initiation proteins between leaves and tuber (Figure 1). Not only do tubers have almost no MRC expression but they also have relatively low SS4 expression, which is consistent with the loss of SS4 interaction in PTST2b (Figure 2). It will be also important to investigate the role of PTST2a and PTST2b in leaves. In leaves, the transcript abundance of PTST2a was much higher than PTST2b (Figure 1b). We speculate that PTST2a could play a major role in starch granule initiation in leaves since its biochemical function in interacting with SS4 is more consistent with that described for Arabidopsis PTST2 (Seung *et al*., 2017). Further analysis of leaf chloroplasts could reveal if PTST2b has any role in determining the number and/or shape of starch granules in chloroplasts.

### Granule morphogenesis in potato requires specific proteins

Our finding that PTST2b is required for granule morphogenesis demonstrates that even granules with apparently ‘simple’ shapes require specific proteins for their morphology. This is consistent with a recent study that demonstrated PHO1a is required for normal granule morphology in potato tubers (Sharma *et al*., 2023), whereby knockout mutants of PHO1a had smaller, rounder granules than the wild type. The fact that silencing PTST2b or eliminating PHO1a both lead to more spherical granules suggests that the two proteins may act in the same morphogenesis process in tubers. Also, the abundance of PTST2 associated with starch granules increases in tubers of PHO1a silencing lines, hinting at a functional link between the proteins (Castellanos *et al*., 2023). We recently reported such a functional interaction in the wheat endosperm, where PTST2 (BGC1) interacts with the ortholog of PHO1 (PHS1), and both proteins are essential for the initiation of the small B‐type granules during grain development (Kamble *et al*., 2023). However, unlike in wheat, we did not detect an interaction between either PTST2 isoform and PHO1a in our pairwise immunoprecipitation assays (Figure 2). This suggests that in potatoes, the two proteins may act together in granule morphogenesis without physical interaction, or that we were not able to detect the interaction using our experimental method. For example, the interaction may be too weak or transient to be detected in our immunoprecipitation assay, or the interaction might require conditions provided by tuber amyloplasts that are not provided by chloroplasts of *N. benthamiana* leaves. It is also possible that another protein might be required to facilitate the interaction, which was not present in our assay. Therefore, future work should prioritize detecting PTST2b interaction partners in the native tuber environment using techniques such as immunoprecipitation and proximity labelling, which could provide more information into the mechanism by which PTST2b influences granule morphology.

However, it should be noted that PTST2b appears to have a more specific role in starch granule morphogenesis than PHO1a. PHO1a has also an established role in maltooligosaccharide (MOS) metabolism in tubers. Tuber discs of PHO1a silencing lines accumulate MOS, and cannot effectively utilize imported glucose‐1‐phosphate for further MOS synthesis (Flores‐Castellanos and Fettke, 2023). Perhaps due to its multiple roles, the loss of PHO1 led to other phenotypes beyond granule shape: including reduced granule size, the accumulation of many small granules, and altered tuber morphology (Sharma *et al*., 2023). None of these phenotypes were observed after PTST2b silencing. Especially, the fact that *pho1a* mutants have smaller granules than the wild type is a key distinction to siPTST2b lines, and warrants future quantitative comparisons of granule morphology between the two lines. This is important considering that our imaging flow cytometer analyses showed that small granules are round even in the wild type, and granules become more ellipsoid as they get larger. The PTST2b silencing lines break this relationship between granule size and shape, as the granules remained round regardless of their size.

As to why such active morphogenesis mechanisms exist in the tuber, and whether there is an adaptive advantage of having ovoid versus spherical granules, remain as pertinent questions. We are not aware of any natural occurrence of spherical granules – in most other root/tuber crops, granules are ovoid (Jane *et al*., 1994). Further experiments, such as the analysis of tuber yields in siPTST2b lines in a field setting, as well as the analysis of starch degradation rates during tuber sprouting, may shed light on the physiological relevance of specific granule shapes.

### MRC overexpression induces fused granules with multiple initiation points

Whilst siPTST2b granules were more rounded than those of the wild type, we also produced the opposite effect by overexpressing MRC. Starch granules of MRC‐OE lines had altered shape parameters, including reduced circularity and aspect ratio values relative to the wild type (Figure 5). Unlike PTST2b silencing, however, we suggest that the reason for the altered granule morphology in MRC‐OE lines is primarily due to an increased number of granule initiations, rather than a direct role for MRC in starch granule morphogenesis. Up to 12% of the starch granules in MRC‐OE lines had multiple initiation points (hila) visible under polarized light, and the hila appeared to have fused to form irregularly shaped granules (Figure 7). Granules with multiple hila were a rare occurrence in the wild type (<2% of total granules observed). In Arabidopsis leaves, MRC promotes starch granule initiation, since knockout mutants in *mrc* have only a few starch granules per chloroplast (Seung *et al*., 2018). The granules with multiple hila in MRC‐OE lines suggest that MRC can similarly promote the number of granule initiations in potato tuber amyloplasts. Granules arising from fused, multiple initiations have been previously observed in potatoes following the mutations of branching enzymes (Tuncel *et al*., 2019) and isoamylase (Bustos *et al*., 2004). It is possible that increasing the number of granules initiated within the confines of an amyloplast leads to the eventual fusion of the granules as they grow, in a similar manner to compound starch granules in grasses (Matsushima *et al*., 2015).

### Applications of potato starch modification by targeting granule morphogenesis

We demonstrated that PTST2b is a gene target for altering the shape of potato starch granules, and that this is an effective approach to alter functional properties of starch without affecting tuber yield or starch content. The siPTST2b lines had a unique pasting profile on the RVA, with increased peak, breakdown and final viscosities (Figure 10). The siPTST2b and MRC OE lines had in common minor changes in amylopectin chain length structure, and a small decrease in amylose content (Figure S9). The reduction in amylose content in siPTST2b is consistent with PTST2 mutants of rice and wheat (Kamble *et al*., 2023; Peng *et al*., 2014; Zhang *et al*., 2021). However, the altered pasting profile was only observed in the siPTST2b lines and not in the MRC‐OE lines, suggesting it is most likely a direct consequence of the round granule shape rather than the altered granule size. Further, the degree to which peak viscosity increased amongst siPTST2b lines was positively correlated to the degree of roundness measured in the granules (Figures 5 and 10). Future research should focus on the mechanism by which granule shape affects pasting behaviour. It is possible that shape affects the interaction between swollen granules during gelatinization, influencing the viscosity. Alternatively, the shape might affect other starch parameters that we did not quantify in this study, such as phosphate content, which could also contribute to viscosity.

The alteration in granule shape in both lines, as well as the change in granule size in MRC‐OE lines, also affects the surface area‐to‐volume ratios of the starch granules. For example, spherical starch granules have the minimum surface area for a given volume of granule and therefore could have reduced digestion rates – similar to the phenomenon observed in large starch granules (Dhital *et al*., 2010). By contrast, the small, irregular granules observed in MRC‐OE lines could increase the relative surface area, increasing digestion rates and making it more suitable for use as a binder in paper and plastics (Lindeboom *et al*., 2004). They may also have lower swelling power, which could make them more suited to extrusion than normal potato starch (Liu, 2005). Systematic testing of these novel starches is now required to identify the various food and industrial applications where the properties of these novel starches can be beneficial.

## Experimental procedures

### Plant transformation and growth

For the gene‐silencing construct used for the siPTST2b lines, we cloned the pSIM2259 construct, which contains an inverted repeat targeting the terminator region of the PTST2b transcript to avoid cross‐reactivity with PTST2a. For MRC overexpression lines, the pSIM2256 construct was cloned. *Solanum tuberosum* genomic DNA from variety Clearwater Russet was isolated by the DNeasy Plant Mini Kit (Qiagen), and 499 bp PTST2b fragments (corresponding to coordinates 1810–2308 of Soltu.DM.01G008470.1) and the MRC coding sequence were amplified using the Q5® High‐Fidelity Polymerase (NEB) using the primers in Table S2. PCR products were gel purified with the QIAquick Gel Extraction Kit (Qiagen) and ligated to the pCR™ Blunt II‐TOPO® vector (Invitrogen). Sequences were confirmed by Sanger sequencing. The PTST2b silencing cassette or MRC coding sequence was placed downstream of the Cauliflower mosaic virus (CaMV) 35S promoter and upstream of the ubiquitin‐3 (UBI3) terminator by a standard restriction enzyme method. Control binary vector (pSIM4030) was used to produce empty vector tissue culture plants. All binary vectors include a mutant acetolactate synthase (mStALS) selectable marker, controlled by the ubiquitin‐7 (UBI7) promoter and nopaline synthase (NOS) terminator. The binary vectors were transferred into *Agrobacterium tumefaciens* strain AGL1, for plant transformation into Clearwater Russet. We isolated at least 25 independent transformants for each construct using PCR primers specific to the T‐DNA insert. In addition, plants transformed with an empty vector (pSIM4030) were isolated as controls.

The transgenic potato lines were grown in glasshouses at Simplot Plant Sciences (Boise, Idaho, USA). Tissue culture plants were grown in Sunshine mix‐1 (www.sungro.com) in two‐gallon pots in a greenhouse controlled for temperature (18 °C minimum/27 °C maximum) and light (16 h photoperiod with an intensity of about 1500 μmol/m^2^/s). Mature tubers were harvested after 3 months of growth, peeled and lyophilized before grinding into flour using metal beads in a Genogrinder ball mill (SPEX).

### Analysis of gene expression by RT‐qPCR

Leaves and small tubers (~1–2 cm diameter) were harvested at the end of the day from glasshouse‐grown Clearwater Russet plants and were flash frozen in liquid N_2_. The tissue was ground into a fine powder using a mortar and pestle, and RNA was extracted using the RNeasy Plant RNA purification kit (Qiagen). cDNA was synthesized using the GoScript Reverse Transcriptase kit (Promega). qPCR was conducted with the SYBR Green JumpStart Taq ReadyMix (Merck) using the primers in Table S2. Relative expression was calculated using the Pffafl method against the *Sec3* reference gene (Tang *et al*., 2017), which was stably expressed between leaves and tubers (Figure S10).

### Transient expression of granule initiation proteins for localization and pairwise immunoprecipitation

The coding sequences of PTST2a and PTST2b were ordered as a gBlocks DNA fragment (IDT DNA) flanked by attB Gateway recombination sites. These coding sequences were recombined into the Gateway entry vector, pDONR221, using the BP Clonase II kit (Thermo Scientific) according to the manufacturer's instructions. The coding sequence of PHO1a was amplified from total cDNA prepared from Clearwater Russet tubers with a 5′CACC overhang (Table S2), and was inserted into the Gateway entry vector, pENTR using the pENTR/D‐TOPO cloning kit (Thermo Scientific). The coding sequences of MFP1 and SS4, flanked with attL recombination sites, were synthesized into the pUC57 vector by GenScript. The coding sequences in pDONR, pENTR or pUC57 vectors were recombined into pUBC‐YFP (for Arabidopsis UBQ10 promoter expression and C‐terminal YFP fusions) or pJCV52 (for CaMV 35S‐driven expression and C‐terminal HA‐tag fusions). All vectors were confirmed using Sanger sequencing before use.

*Nicotiana benthamiana* leaves were transiently transformed by infiltrating *Agrobacterium* cultures harbouring the relevant constructs, and pairwise immunoprecipitations using anti‐GFP/YFP microbeads (Miltenyi Biotech) were carried out as previously described (Kamble *et al*., 2023). Confocal laser scanning microscopy was carried out using the Leica Stellaris 8 laser‐scanning confocal microscope and a 40x water immersion objective. YFP signal was excited using a white light laser set to 514 nm and emission was detected at 562–623 nm. Chlorophyll autofluorescence was excited at 555 nm and emission was detected at 767–822 nm (Esch *et al*., 2023).

### Starch granule purification

Starch granules were extracted in 96‐well deep‐well plates from potato flour as described by Thieme *et al*. (2023), with a few modifications. The flour was incubated with Viscozyme (Merck) in sodium acetate buffer (0.1 M, pH 4.8) at 42 °C for 16 h to digest cell walls. The digest was then spun through a Percoll (Merck) cushion at 2500 *g*, 10 min to separate the starch pellet from the cell debris. The starch pellets were washed twice with water, before final resuspension in water for further analysis.

### Granule size analysis by flow cytometry (FC) and Coulter counter (CC)

For analysis by flow cytometry, the purified starch granule suspension was analysed on an LSR Fortessa flow cytometer (BD) using the 488 nm laser and the following settings: FSC‐A voltage: 100, SSC‐A voltage: 150, FSC‐A threshold: 800, FSC ASF: 0.2. The flow rate was between 3000 and 6000 events per second until 50 000 events per sample were recorded. Size reference beads of poly(methyl methacrylate) (PMMA, Sigma–Aldrich) with diameters of 1, 8, 30 and 60 μm and a refractive index of 1.497 at 488 nm (Sultanova *et al*., 2009) were used to correlate FSC with particle diameter. The histograms and gates were created using FloJo V.10.

For analysis by the Coulter counter, purified starch granules were analysed on the Multisizer 4e Coulter counter (Beckman Coulter), fitted with a 200 μm aperture. A minimum of 100 000 particles were analysed per sample. Data for relative volume vs. diameter plots were collected using linear bin spacing.

### Image flow cytometry (iFC)

The purified starch suspension was analysed using an Amnis ImageStream MKII image flow cytometer. The acquisition settings were as follows: core size of 10 μm, flow speed of 132.0 mm/s, and LED intensity of 17.48 mW for brightfield illumination. The autofocus function was disabled to expedite acquisition, aiming for approximately 300 objects per second within the selected singlet gate. Data acquisition was completed after 20 000 events, and subsequent analysis was performed using IDEAS 6.2 software. In the analysis software, a mask was created based on intensity values ranging from 0 to 600. The spot count feature was used to filter out images with more than one spot. In a second round of filtering, a more stringent intensity range of 0–13 was applied to the spot count feature. However, for further analysis, the intensity range of the mask was reverted to 0–600. This adjustment allowed for the utilization of shape parameters, as depicted in Figure S6, to remove closely attached starch granules. The final result was an image dataset predominantly consisting of individual starch granules. These images have been used to calculate the circularity and aspect ratio with the same software.

### Microscopy observations of starch granules

For observing the morphology of starch granules using SEM, purified granules were dried onto a glass cover slip and mounted onto an SEM stub. Samples were sputter coated with 5 nm platinum using an ACE 600 sputter coater (Leica), before imaging on a Nova NanoSEM (FEI). For imaging of growth rings in fractured granules, potato flour was ground for an additional 5 min on the Genogrinder, before extracting and imaging as described.

For polarized light microscopy, purified granules in 40% glycerol (v/v) were mounted onto a cover slip and imaged on an Imager.Z2 microscope fitted with NEC Plan‐NEOFLUAR 10× and 20× lenses and a cross‐polarization filter (Carl Zeiss).

For observing starch granules in potato parenchyma tissue, small cubes of tissue from freshly harvested mature tubers were fixed in 2.5% glutaraldehyde in 0.05 M sodium cacodylate, pH 7.4, which was vacuum infiltrated into the tissue. The segments were post‐fixed with osmium, dehydrated in an ascending ethanol series, and embedded in LR white resin using an EM TP embedding machine (Leica). Semi‐thin sections (0.5 μm thick) were produced from the embedded leaves using a glass knife and were dried onto PTFE‐coated slides. Sections were stained using toluidine blue stain (0.5% toluidine blue ‘O', 0.5% sodium borate) for 1 min before mounting in Histomount (National Diagnostics). Sections were imaged on an Imager.Z2 microscope fitted with an NEC Plan‐NEOFLUAR 20x lens (Zeiss).

### Starch content

We assayed starch as the amount of glucose released following starch hydrolysis. In a 96‐well format, potato flour (2 mg) was mixed with 110 mM sodium acetate buffer (pH 4.8, 200 μL), before digesting it with α‐amylase and amyloglucosidase (Megazyme, Total Starch Assay Kit). After incubation and centrifugation, the supernatant was transferred to a new plate and diluted. Glucose was quantified using a hexokinase/glucose‐6‐phosphate dehydrogenase‐based assay (Roche).

### Amylose content

Amylose content was analysed by mixing approximately 1 mg of extracted starch with 200 μL of water. Subsequently, 200 μL of 2 M NaOH was added to the starch solution and left to incubate overnight at room temperature. The NaOH was then neutralized by adding 400 μL of 1 M HCl, and the pH was checked using pH strips.

The solution (8 μL) was combined with 217 μL of water and 25 μL of Lugol solution in 96‐well microtitre plates. Three technical replicates were prepared for each sample, including the blank. Following a 10‐min incubation at room temperature, the absorbance of the samples was measured at 620 and 535 nm using a spectrophotometer. The absorbance ratio was calculated as (620 nm − blank)/(535 nm − blank). To estimate the amylose to amylopectin ratios were calculated using the formula: Percentage amylose = (3.5 − (5.1 × A_618_/A_550_))/((10.4 × A_618_/A_550_) − 19.9). The selection of wavelengths 618 nm and 550 nm was based on the study by Hovenkamp‐Hermelink et al. (1988).

### Moisture content

Two slices were taken from each of the three individual tubers, and all six slices were combined for one sample. Tuber slices were diced, collected in pre‐weighed 15 mL tubes (W_tube_), and weighed prior to flash freezing in liquid Nitrogen (W_wet_). Samples were placed in the freeze dryer for 3 days. Following freeze‐drying, sample tubes were weighed (W_dry_) and the percent moisture was calculated using the following formula: Moisture content (%) = (W_wet_ – W_dry_)/(W_wet_ – W_tube_) × 100.

### Amylopectin chain length distribution

Amylopectin chain length distribution was analysed using High‐Performance Anion Exchange Chromatography with Pulsed Amperometric Detection (HPAEC‐PAD) on a Dionex ICS‐5000‐PAD fitted with a PA‐100 column (Thermo). Debranched starch samples were prepared directly from lyophilized potato flour, using the method described in Streb *et al*. (2008).

### Rapid Visco analysis

Rapid visco analysis (RVA) was carried out on an RVA Tecmaster (Perten) running a modified AACC general pasting method (76–21), where the hold temperature at 97 °C has been extended to 18 min. Analyses were performed with 1.5 g of purified potato starch in 25 mL of water.

### Phylogenetic analyses

For constructing phylogenetic trees, we used BLASTp to obtain amino acid sequences with homology to the Arabidopsis granule initiation proteins (SS4, PTST2, PTST3, MRC, MFP1 and SS5) from Solanaceae and diverse plant species, using the SolGenomics and Phytozome databases (Fernandez‐Pozo *et al*., 2015; Goodstein *et al*., 2012). Sequences from *Solanum chacoense* and *Solanum candolleanum* were obtained from SpudDB (Hirsch *et al*., 2014). The sequences were aligned using MAFFT (Katoh and Standley, 2013), and were manually trimmed to include regions with good alignment (trimmed alignments are provided as File S1). Trees were constructed using the Maximum Likelihood method using RaxML (Edler *et al*., 2020), using the Blosum62 substitution model and 1000 bootstrap replicates.

## Author contributions

D.S. conceived the study. D.S. and J.W.H designed the study. All authors performed the research and analysed data. A.H and D.S. wrote the article with input from all authors.

## Conflict of interest

A.H. and D.Se. are co‐inventors of a patent for using PTST2b to modify starch. D.Se. is an inventor of a patent for using MRC to modify starch. M.C, A.R, D.Sc. and J.W.H are employed by the J.R. Simplot Company that develops biotech potato crops.

## Accession numbers

The accession numbers for the genes investigated in this study are PTST2a (Soltu.DM.05G006480), PTST2b (Soltu.DM.01G008470) and MRC (Soltu.DM.08G009720).

## Supporting information


**Figure S1.** Phylogenetic analysis of potato granule initiation proteins.
**Figure S2.** Amino acid sequence alignment of PTST2a and PTST2b isoforms.
**Figure S3.** Screening of siPTST2b transgenic lines on the flow cytometer.
**Figure S4.** Screening of MRC OE transgenic lines on the flow cytometer.
**Figure S5.** Quantification of granule size distribution using flow cytometry gates.
**Figure S6.** Workflow for granule morphology analyses on the ImageStream.
**Figure S7.** Transcript levels of PTST2b and MRC in siPTST2b and MRC‐OE tubers.
**Figure S8.** Quantification of PTST2a transcript in siPTST2b lines.
**Figure S9.** Analysis of starch polymer composition and structure in the transgenic lines.
**Figure S10.** Comparison of qPCR reference genes for leaf and tuber tissues.
**Table S1.** Potato orthologs of the known granule initiation proteins.
**Table S2.** Primers used in this study.
**Table S3.** Mean granule circularity and aspect ratio values for the lines analysed in Figure 5.

**File S1.** Alignments used for phylogenetic analyses.

## Data Availability

The data that support the findings of this study are available in the main figures and in the Supplementary Material.
