# Supplementary material for: Distinct effects of PTST2b and MRC on starch granule morphogenesis in potato tubers
Source: Plant Biotechnol J. 2024 Dec 10;23(2):412–29. doi: 10.1111/pbi.14505 (PMC11772324; doi:10.1111/pbi.14505)
Supplement: Supplementary file 1 — Figure S1. Phylogenetic analysis of potato granule initiation proteins. Figure S2. Amino acid sequence alignment of PTST2a and PTST2b isoforms. Figure S3. Screening of siPTST2b transgenic lines on the flow cytometer. Figure S4. Screening of MRC OE transgenic lines on the flow cytometer. Figure S5. Quantification of granule size distribution using flow cytometry gates. Figure S6. Workflow for granule morphology analyses on the ImageStream. Figure S7. Transcript levels of PTST2b and MRC in siPTST2b and MRC‐OE tubers. Figure S8. Quantification of PTST2a transcript in siPTST2b lines. Figure S9. Analysis of starch polymer composition and structure in the transgenic lines. Figure S10. Comparison of qPCR reference genes for leaf and tuber tissues. Table S1. Potato orthologs of the known granule initiation proteins. Table S2. Primers used in this study. Table S3. Mean granule circularity and aspect ratio values for the lines analysed in Figure 5. [file PBI-23-412-s001.docx]

**Hochmuth et al. Supplemental Figures and Tables**

**A**

1

**B**

**C**

2

**D**

**Supplemental Figure 1: Phylogenetic analysis of potato granule initiation proteins.** Maximum likelihood phylogenetic trees were produced for SS4 and SS5 (A), PTST3 (B), MRC (C) and MFP1 (D) using RaxML with 1000 bootstrap replicates. Bootstrap values >50 are shown next to the nodes. Branch lengths represent the number of substitutions per site, indicated by the scale bars.

3

4

**Supplemental Figure 2: Amino acid sequence alignment of PTST2a and PTST2b isoforms.** Full-length sequences from *Solanum tuberosum* [*St*PTST2a (Soltu.DM.05G006480.1) and *St*PTST2b (Soltu.DM.01G008470.1)], *Solanum candolleanum* [*Sca*PTST2a (Solca.05G005750.1) and *Sca*PTST2b (Solca.01G008400.1)] and *Solanum chacoense* [*Sch*PTST2a (Solch.M6.05G006650.3) and *Sch*PTST2b (Solch.M6.01G009440.6)] were aligned with the sequence of *Arabidopsis thaliana At*PTST2 (AT1G27070.1). Alignments were performed with MAFFT and visualised with ESPript. The transit peptide, coiled coil and carbohydrate binding module 48 domains are marked underneath the sequence alignment. Strictly conserved identical residues are depicted with white letters on a red background; residues with at least 70% conservation (based on similarity of side chains) are in red text on a white background, while those with less than 70% conservation are in black text on a white background. All conserved residues are framed in blue.

5

**Supplemental Figure 3: Screening of siPTST2b transgenic lines on the flow cytometer.** Tuber starch was extracted from the first generation of transgenic lines grown in the glasshouse. The forward scatter trace for the WT control (blue trace) is shown with the forward scatter trace for each transgenic line (red trace). The stars indicate selected lines for further analysis in a subsequent glasshouse generation.

6

**Supplemental Figure 4: Screening of MRC OE transgenic lines on the flow cytometer.** Tuber starch was extracted from the first generation of transgenic lines grown in the glasshouse. The forward scatter trace for the WT control (blue trace) is shown with the forward scatter trace for each transgenic line (red trace). The stars indicate selected lines for further analysis in a subsequent glasshouse generation.

7

**Supplemental Figure 5: Quantification of granule size distribution using flow cytometry gates.** The size gate analyses are the same as those presented in Figure 3B, but all lines are presented separately. Each panel shows the size range indicated on the top right corner. Values with different letters are significantly different using a one-way ANOVA and Tukey test at p < 0.05.

8

**Supplemental Figure 6: Workflow for granule morphology analyses on the ImageStream. A)** Representative panel of micrographs obtained for each transgenic lines, before and after filtering to remove images of insufficient quality for analysis of shape parameters (e.g: images containing multiple granules). **B)** Procedure for automated image filtering based on spot count and shape ratio feature. The first round of filtering by spot count removes images containing multiple separated granules. A more stringent mask was then used to separate starch granules that were close to each other, resulting in two spot counts, and these images were subsequently excluded. In the last step, the shape ratio parameter ratio between thickness and length were analysed, thus filtering out starch granules that were very close together or overlap. **C)** Numbers of images acquired and analysed in the analyses of Figure 5, after automated image filtering steps.

9

**Supplemental Figure 7: Transcript levels of PTST2b and MRC in siPTST2b and MRC-OE tubers.** RT-qPCR was used to quantify transcript levels in developing tubers from eight different transgenic lines (indicated with different numbers) and the empty vector (EV) control. Values are means ± SEM of n=2-6 replicates. Red arrows indicate selected lines for detailed analyses.

10

**Supplemental Figure 8: Quantification of PTST2a transcript in siPTST2b lines.** RT-qPCR was used to quantify transcript levels in mature tubers from the siPTST2b lines (indicated with different numbers) and the empty vector (EV) control. Values are means ± SEM of n=3-8 replicates. For both panels, values that are significantly different to the empty vector (EV) control under a pairwise two-tailed t-test (at p < 0.05) are marked with an asterisk (*)

11

**Supplemental Figure 9: Analysis of starch polymer composition and structure in the transgenic lines. A)** Apparent amylose content of tuber starch from transgenic lines. **B)** Chain length distribution of amylopectin determined on debranched tuber starch using High Performance Anion Exchange Chromatography with Pulsed Amperometric Detection (HPAEC-PAD). **C)** Principal component analysis of chain length distributions. The four transgenic lines were plotted separately and enclosed with ellipses. The arrows represent the redundancy analysis, showing the major chain lengths driving the separation on the plot.

12

**Supplemental Figure 10: Comparison of qPCR reference genes for leaf and tuber tissues.** Raw Ct values obtained using primers against four reference genes (EF1a, Sec3, Tubulin and Actin) that are described in Tang et al., (2017). Values represent means ±SEM from n=3 biological replicates (each replicate represents a separate plant). Only the Actin primers (marked with an asterisk) showed a significant difference in Ct values between leaf and tuber samples, using a two-tailed t-test at p < 0.05.

13

**Supplemental Table S1. Potato orthologs of the known granule initiation proteins. Expression**

**Gene** **Accession number** **Leaf** **Stolon** **Young** **Mature tuber tuber**

MFP1 PGSC0003DMG400002564 **4.16** 2.5 3.12 1.75 MRC PGSC0003DMG400004306 **12.31** 0.33 0.00 0.00 PTST2a PGSC0003DMG400017617 **0.47** 0.20 0.17 0.05 PTST2b PGSC0003DMG400026781 0.30 1.11 **1.74 1.70** PTST3 PGSC0003DMG401003864 1.47 1.64 **3.27** 1.52 SS4 PGSC0003DMG400008322 2.01 **4.80** 2.76 1.38

SS5 PGSC0003DMG400030619 0.42 4.75 14 **19.15** Expression data from a diploid potato variety available on the Potato eFP browser. Values represent absolute transcript levels, normalised by the GCOS method, with a TGT value of 100. The highest expression level for each gene is highlighted in bold.

**Supplemental Table S2. Primers used in this study. Primers for RT-qPCR**

**Gene** **Forward primer** **Reverse primer** **Primer target efficiency**

**(%)** MFP1 CAGAAAGAGGAAACAGAGCAAAC CGAGCCAAGGCATAAAGAGA 94.4 MRC CCAGCCAACGGAGTTCTG ACCTGACAATTCTTGAGGAAG 93.4

G

PTST2a GCTTTACAGTTTCTTCGCACTAC CTCCATCTTCCTCTGAGTTGT 85.7 C

PTST2b CGTCCTCTGGTAGATTGATAGAAAC ACGCTTTCCCAGATCAATACC 98.4 PTST3 CACAGAACGGAAGGCAAGA GCACTCATAGGTTGGTTGTTT 93.2

G

SS4 GCGGCCTTACTCAGATGATAG GGGATTGAGTCATCGTCAAC 108.0 A

SS5 GACATCATGCTATGCCCTTCT CCACAGAGTGCCCGTATTT 101.1 Sec3 GCTTGCACACGCCATATCAAT TGGATTTTACCACCTTCCGCA 100.9 **Primers for cloning PTST2b fragment for the pSIM2259 construct**

**Gene** **Forward primer** **Reverse primer target**

PTST2b CACAATGATGGACACGAAAA AGAGGACGACGAAAAAGA **Primers for cloning MRC CDS for the pSIM2256 construct**

**Gene** **Forward primer** **Reverse primer target**

MRC ATGGCGTTGCCAGCCTTACCGCGCGC CTACTGGGGGCATAAAATAC CACTTTATCGTTTTCCTCTCTTTGCCAG CCAACGGAGTTCTGTTTTATGAGGCTC

GAATGGAAG

**Primers for cloning PHO1a cDNA with attB sites**

**Gene** **Forward primer** **Reverse primer target**

PHO1a CACCATGGCGACTGCAAATGGAGC TGCTATTTCCACAGCTTCAATGTTCCA All primer sequences are given from 5’ to 3’ end. MRC first exon sequence included in the forward primer is underlined. The Sec3 primers are from Tang *et al.* (2017). The “CACC” sequence required for directional D-TOPO cloning of PHO1a is underlined.

14

**Supplemental Table S3. Mean granule circularity and aspect ratio values for the lines analysed in Figure 5.**

Mean Circularity Mean Aspect Ratio

CW Empty 1 13.61 0.862 CW Empty 2 12.44 0.839 CW Empty 3 11.78 0.831 Mean 12.61 0.844

siPTST2b 02 21.82 0.938 siPTST2b 07 20.54 0.940 siPTST2b 15 21.60 0.946 siPTST2b 17 19.04 0.944 Mean 20.75 0.942

p value (t-test to CW Empty) 0.0002 0.0001

MRC OE 11 10.40 0.815 MRC OE 12 10.76 0.829 MRC OE 15 10.73 0.826 MRC OE 18 10.51 0.840 Mean 10.60 0.828

p value (t-test to CW Empty) 0.0072 0.1555

15
